# Meta-analysis of organ-specific differences in the structure of the immune infiltrate in major malignancies

**DOI:** 10.18632/oncotarget.4180

**Published:** 2015-05-19

**Authors:** Gautier Stoll, Gabriela Bindea, Bernhard Mlecnik, Jérôme Galon, Laurence Zitvogel, Guido Kroemer

**Affiliations:** ^1^ Université Paris Descartes, Sorbonne Paris Cité, Paris, France; ^2^ Equipe 11 labellisée Ligue Nationale contre le Cancer, Centre de Recherche des Cordeliers, Paris, France; ^3^ Institut National de la Santé et de la Recherche Médicale, Paris, France; ^4^ Université Pierre et Marie Curie, Paris, France; ^5^ Metabolomics and Cell Biology Platforms, Gustave Roussy Cancer Campus, Villejuif, France; ^6^ Institut National de la Santé et de la Recherche Médicale, Equipe labellisée Ligue Nationale Contre le Cancer, Villejuif, France; ^7^ Institut Gustave Roussy Cancer Campus, Villejuif, France; ^8^ Faculty of Medicine, University of Paris Sud, Kremlin-Bicêtre, France; ^9^ Center of Clinical Investigations in Biotherapies of Cancer (CICBT), Villejuif, France; ^10^ Pôle de Biologie, Hôpital Européen Georges Pompidou, AP-HP, Paris, France

**Keywords:** meta-analysis of microarrays, breast cancer, colorectal carcinoma, melanoma, non-small cell lung cancer

## Abstract

Anticancer immunosurveillance is one of the major endogenous breaks of tumor progression. Here, we analyzed gene expression pattern indicative of the presence of distinct leukocyte subtypes within four cancer types (breast cancer, colorectal carcinoma, melanoma, and non-small cell lung cancer) and 20 different microarray datasets corresponding to a total of 3471 patients. Multiple metagenes reflecting the presence of such immune cell subtypes were highly reproducible across distinct cohorts. Nonetheless, there were sizable differences in the correlation patterns among such immune-relevant metagenes across distinct malignancies. The reproducibility of the correlations among immune-relevant metagenes was highest in breast cancer (followed by colorectal cancer, non-small cell lung cancer and melanoma), reflecting the fact that mammary carcinoma has an intrinsically better prognosis than the three other malignancies. Among breast cancer patients, we found that the expression of a lysosomal enzyme-related metagene centered around *ASAH1* (which codes for N-acylsphingosine amidohydrolase-1, also called acid ceramidase) exhibited a higher correlation with multiple immune-relevant metagenes in patients that responded to neoadjuvant chemotherapy than in non-responders. Altogether, this meta-analysis revealed novel organ-specific features of the immune infiltrate in distinct cancer types, as well as a strategy for defining new prognostic biomarkers.

## INTRODUCTION

Ever accumulating preclinical and clinical evidence indicates that anticancer immunosurveillance is (one of) the most important factor(s) that naturally (i.e., in the absence of therapy) limits tumor progression and that determines the efficacy of conventional therapeutic interventions including chemotherapy with cytotoxic drugs and radiotherapy. This general rule applies to most if not all cancers including, but not limited to, breast cancer [1], colorectal carcinoma [2, 3], melanoma [4] and non-small cell lung cancer [5, 6].

As a broad principle, it appears that the presence of cytotoxic T lymphocytes, especially if they have a memory effector T cell phenotype [7-9], as well as that of conventional dendritic cells, has a positive prognostic impact on cancer patients, in particular if such cells are found within tumor nodules instead of the surrounding stroma [10]. Moreover, the intratumoral presence of tertiary lymphoid organs, which are elaborate micro-anatomical structures for mounting immune response, has a favorable prognostic value [5, 11]. In contrast, a series of other tumor-infiltrating leukocyte subpopulations has a negative impact. This applies to myeloid-derived suppressor cells, macrophages, M2 macrophages, Th2 helper T cells, and regulatory T cells in several major cancer types [12, 13]. However, in exceptional cases such as renal cell carcinoma [14], head and neck cancer [15] and lymphomas [10], the infiltration by CD8^+^ cytotoxic T lymphocytes may constitute a negative prognostic feature, underscoring different relationships between immunosurveillance and neoplasia in different cancer types.

There are two major technologies to retrieve information on the immune infiltrate. The first technique consists in performing immunophenotyping of infiltrating leukocytes either in situ (by immunohistochemistry or in situ immunofluorescence staining) or on cell suspensions that are generated by mechanic disruption and enzymatic digestion of fresh tumors. Immunophenotyping yields quantitative information on leukocyte subpopulations (and even information on their spatial distribution, if determined in situ), yet is limited to only a few antigens and is poorly standardized [10]. The second technique consists in analyzing the presence of distinct mRNA species within the tumor, usually by microarray or RNAseq technology [16]. This approach, which has the advantage of being highly standardized, yields information on the abundance of different leukocyte subpopulation-specific mRNA species. These results can be ‘deconvoluted’ by specific techniques, for instance by the analysis of certain groups of genes whose co-regulated expression is characteristic of specific leukocyte subtypes, hence constituting a ‘metagene’. More than 30 leukocyte subtypes, each corresponding to a distinct metagene, have been identified in microarray analyses of colon cancers [11, 17].

Recently, we have started the meta-analysis of distinct collections of breast cancer microarrays, observing the existence of immune-relevant metagenes that impact on the therapeutic response to neo-adjuvant chemotherapy [18], although microarrays data do not provide any direct information on the density of the immune infiltrate. Driven by these encouraging results, we decided to perform a systematic analysis of 20 cohorts representing 4 distinct major cancer types with the scope of comparatively determining the composition of the immune infiltrate, as well as the internal correlations among distinct leukocyte subtype-specific metagenes within each cohort. These analyses revealed that the robustness of the ‘structure’ of the immune infiltrate varies in different cancer types and that it likely reflects the efficacy of immunosurveillance.

## RESULTS AND DISCUSSION

### Reproducibility of metagenes corresponding to distinct tumor-infiltrating leukocyte subpopulations

**Figure 1:**
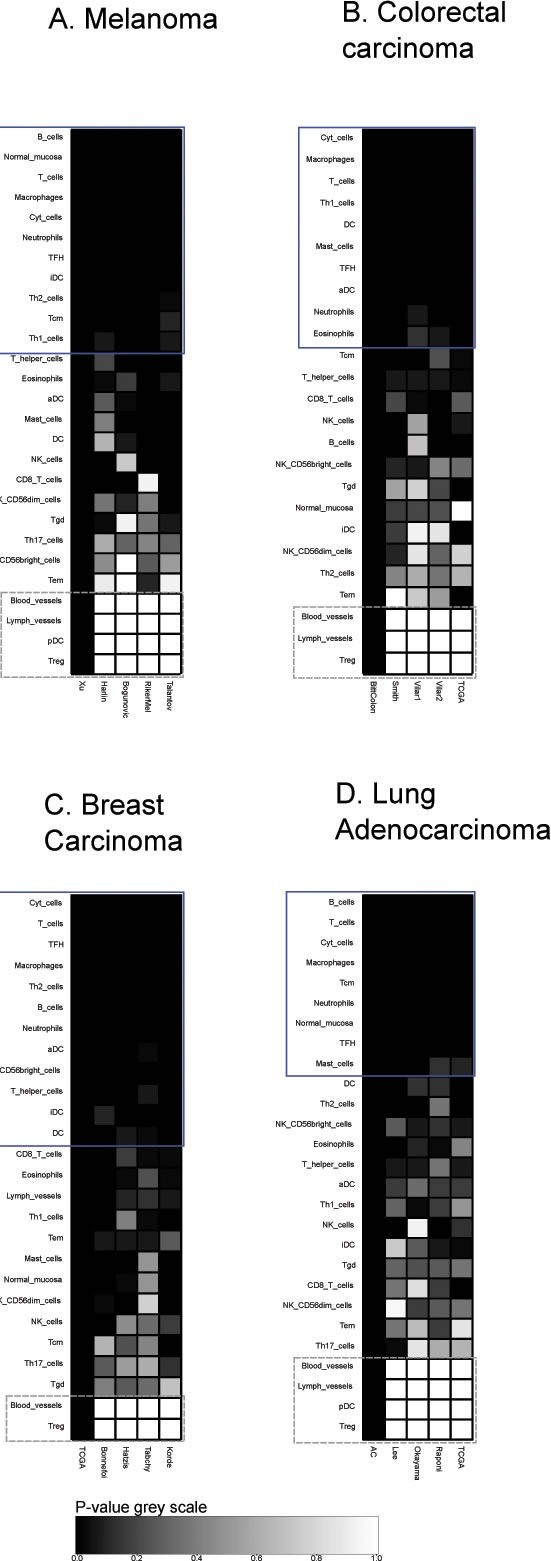
Immune metagene reproducibility Heat maps of reproducibility *p*-values, for each cancer type, for each immune metagene, in each dataset. *P*-values are produced by reproducibility test described in Material and Methods (by definition, reproducibility in the learning dataset has a 0 *p*-value). Blue rectangles represent reproducible metagenes. Grey rectangles represent single-gene metagenes (in that case, reproducibility *p*-value always equals to 1).

**Figure 2:**
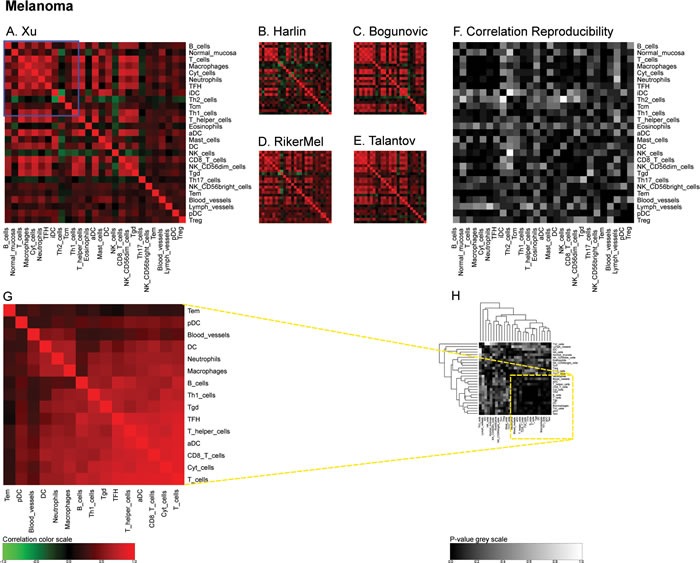
Immune metagene correlations (A-E) and correlation reproducibility *p*-values (F), in melanoma; reproducible correlations of first dataset (G), identified by hierarchical clustering of reproducibility *p*-values (H) Heat map representation of metagene correlation matrices, in the 5 datasets of melanoma transcriptome (blue rectangle corresponds to reproducible metagenes of Figure 1). A correlation reproducibility test is applied to the 5 correlation matrices, producing a matrix of p-values. Clustering of correlation reproducibility (H) allows for the identification of a sub-part of correlation matrix (yellow square), represented for the learning dataset (G).

**Figure 3:**
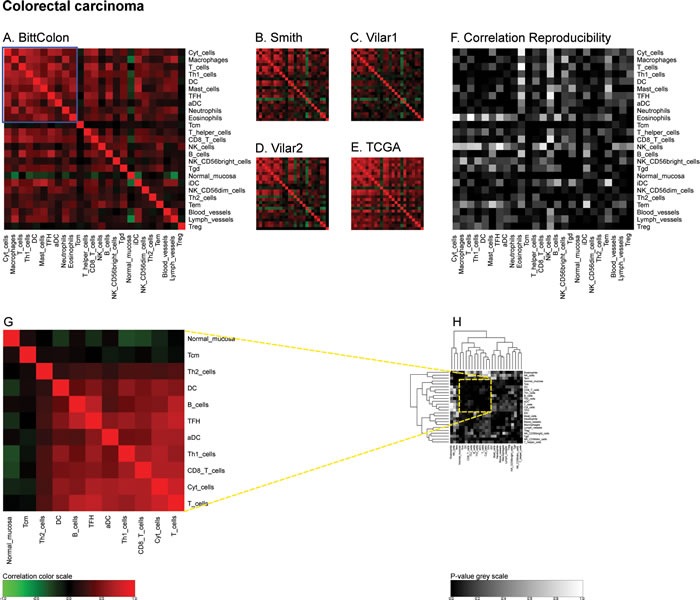
Immune metagene correlations (A-E) and correlation reproducibility *p*-values (F), in colorectal cancer; reproducible correlations of first dataset (G), identified by hierarchical clustering of reproducibility *p*-values (H) Heatmap representation of metagene correlation matrices, in the 5 datasets of melanoma transcriptome (blue rectangle corresponds to reproducible metagenes of Figure 1). A correlation reproducibility test is applied to the 5 correlation matrices, producing a matrix of *p*-values. Clustering of correlation reproducibility (H) allows identifying sub-part of correlation matrix (yellow square), represented for the learning dataset (G).

**Figure 4:**
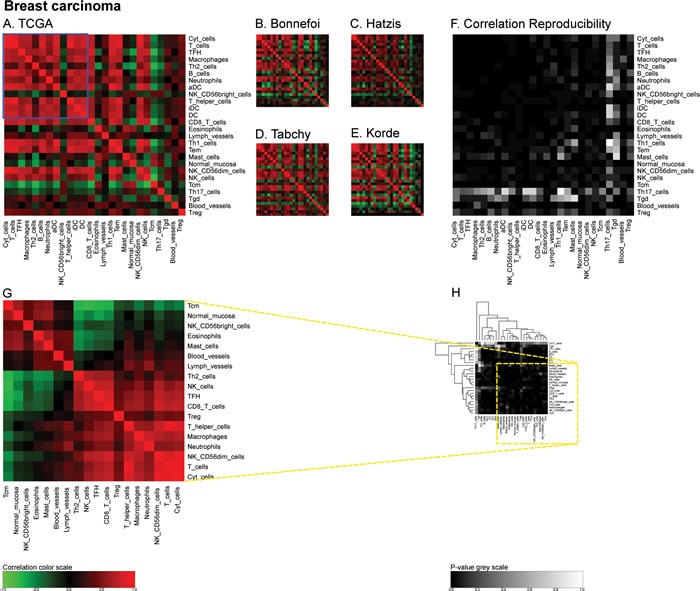
Immune metagene correlations (A-E) and correlation reproducibility *p*-values (F), in breast cancer; reproducible correlations of first dataset (G), identified by hierarchical clustering of reproducibility *p*-values (H) Heatmap representation of metagene correlation matrices, in the 5 datasets of melanoma transcriptome (blue rectangle corresponds to reproducible metagenes of Figure 1). A correlation reproducibility test is applied to the 5 correlation matrices, producing a matrix of *p*-values. Clustering of correlation reproducibility (H) allows identifying sub-part of correlation matrix (yellow square), represented for the learning dataset (G).

**Figure 5:**
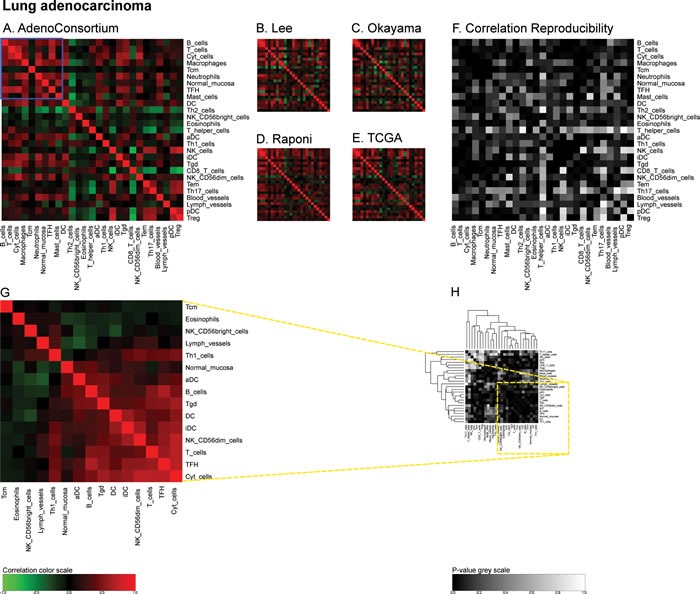
Immune metagene correlations (A-E) and correlation reproducibility *p*-values (F), in lung cancer; reproducible correlations of first dataset (G), identified by hierarchical clustering of reproducibility *p*-values (H) Heatmap representation of metagene correlation matrices, in the 5 datasets of melanoma transcriptome (blue rectangle corresponds to reproducible metagenes of Figure 1). A correlation reproducibility test is applied to the 5 correlation matrices. producing a matrix of *p*-values. Clustering of correlation reproducibility (H) allows identifying sub-part of correlation matrix (yellow square), represented for the learning dataset (G).

A collection of metagenes that correspond to distinct tumor-infiltrating leukocyte subtypes [11] was analyzed for their reproducibility across distinct cohorts of cancers. For each cancer type (breast cancer, colorectal carcinoma, melanoma and lung adenocarcinoma), we chose the cohort comprising the largest number of patients to establish leukocyte-specific metagenes in which the relative contribution of each individual gene was weighted (Table 1). Then, we determined whether this specific metagene could be confirmed in other four cohorts, calculating the *p*-value of reproducibility in each case. While the majority of metagenes were highly reproducible (as this applies for instance for the metagenes indicating the presence of T cells, NK cells, macrophages and neutrophils), a minority was not, including for instance those signifying the presence of regulatory T cells, Tregs, or blood vessels in the tumor, because these metagenes are composed of a single gene (gray squares in Figure 1). Metagenes that were considered as reproducible (by visual inspection) are indicated by the blue squares in Figures 1, 2, 3, 4, 5.

**Table 1:** Characteristics of the 20 patient cohorts treated in this meta-analysis

Cancer type	Cohort name	Number of samples	Characteristics of the cohort	Treatment & outcome	Reference
Melanoma	Xu	83	Primary and metastatic tumors		GSE8401
Melanoma	Harlin	44	Metastatic tumors		GSE12627
Melanoma	Bogunovic	44	Metastatic tumors		GSE19234
Melanoma	RikerMel	56	Primary and metastatic tumors		GSE7553
Melanoma	Talantov	45	Primary tumors		GSE3189
Colon	BittColon	307	Various colon tumors		GSE2109
Colorectal	Smith	177	Various colorectal tumors		GSE17536
Colon	Vilar1	155	Colon tumors		GSE26682, 1st set
Colon	Vilar2	176	Colon tumors		GSE26682, 2nd set
Colon	TCGA	174	Various colon tumors		TCGA consortium
Breast	TCGA	522	Various breast tumors		TCGA consortium
Breast	Bonnefoi	161	Locally advance or large operable breast tumors, estrogen receptor negative	FEC or ET treatment. Pathological complete response (complete disappearance of the tumour with no more than a few scattered tumour cells) *vs* no pathological complete response	GSE6861
Breast	Hatzis	198	HER2 negative breast tumors	Taxane-anthracycline chemotherapy pre-operatively and endocrine therapy if ER-positive. Pathological complete response (no invasive or metastatic breast cancer identified) *vs* rapid development	GSE25065
Breast	Tabchy	178	Various type of breast tumors before treatment	FEC or FAC neo-adjuvant chemotherapy. Pathological complete response *vs* residual disease (clinical or radiological progression)	GSE20271
Breast	Korde	61	Various type of breast tumors, stage 2 or 3 breast cancer with tumor size ≥2cm at patients selection, prior to AC treatment	4 cycles of TX, 4 cycles of adriamycin, cyclophosphamide on day 1 and 21 (neoadjuvant) and AC (neo-adjuvant or adjuvant). Response vs no response (change in tumor size by clinical exam and pathological response).	GSE18728
Lung	AdenoConsortium	462	Various type of Adenocarcinomas		Director's Challenge Lung Study, National Cancer Institute (NHI)
Lung	Lee	138	Adenocarcinoma and squamous cell carcinoma		GSE8894
Lung	Okayama	226	Adenocarcinoma		GSE31210
Lung	Raponi	130	Squamous cell carcinoma		GSE4573
Lung	TCGA	134	squamous cell carcinoma		TCGA consortium

### Correlation of leukocyte subpopulation-associated metagenes in distinct cancer types and cohorts

In the next step, we determined the Pearson's correlation coefficients (R values) for a matrix comprising all immune-related metagenes for the largest melanoma cohort denoted ‘Xu’ according to the name of the first author of the paper describing the cohort [19]. This matrix revealed some strong positive correlations (for instance between CD8 T cells and cytotoxic cells or between T and B cells), which were denoted in red, as well as some negative correlations (for instance between follicular helper T cells [TFH] on one side and B or T cells on the other side), which were denoted in green (Figure 2A). The same correlation matrix was re-calculated for four distinct melanoma cohorts denoted ‘Harlin’, ‘Bogunovic’, ‘RikerMel’, ‘Talantov’ according to the names of the authors describing them [20, 21, 22, 23]. While some correlations including the aforementioned ones are conserved across the cohorts, other positive and negative correlations appeared to be cohort-specific (Figure 2B-2E). We then calculated the reproducibility of the correlations as a *p*-value, meaning that positive or negative correlations, as well as absent correlations, that were observed throughout all cohorts received a low p value, denoted as black or dark grey, while major variations in the correlations received a high p value, denoted as light grey or white (Figure 2F). We then applied the same type of meta-analysis to colorectal cancers in which we calculated a first correlation matrix on the ‘Bitt’ cohort (http://www.intgen.org/) (Figure 3A), re-calculated this matrix for four additional cohorts (Smith [24, 25], Vilar1 & Vilar2 [26-28], TCGA (http://cancergenome.nih.gov/)) (Figure 3B-3E), and determined the reproducibility among such correlations (Figure 3F).

Similarly, we calculated correlation matrices for the ‘TCGA’ breast cancer cohort (http://cancergenome.nih.gov/) (Figure 4A) and four smaller cohorts (Bonnefoi [29], Hatzis [30], Tabchy [31], Korde [32]) (Figure 4B-4E) and the resulting correlation reproducibility (Figure 4F). Finally, we determined immune cell subtype-related correlation matrices for the large ‘AdenoConsortium’ cohort of non-small cell lung cancers [33] (Figure 5A), four additional cohorts (Lee [34], Okayama [35, 36], Raponi [37], TCGA (http://cancergenome.nih.gov/)) (Figure 5B-5E), and their reproducibility (Figure 5F). These correlation matrices were then subjected to a meta-analysis to reveal organ-specific differences in the characteristics of the immune infiltrate that can be deduced from microarray data.

### A meta-analysis of distinct leukocyte and stress response-associated metagenes in distinct cancer types

**Figure 6:**
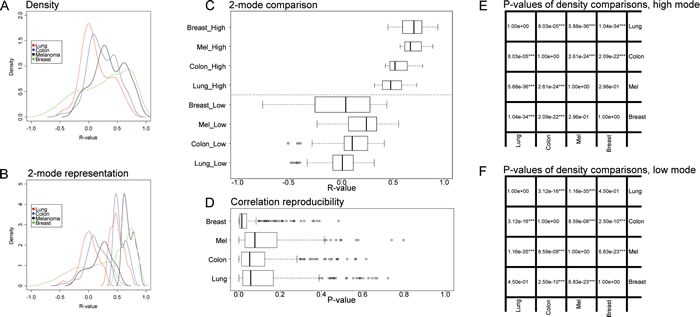
Global pattern of reproducible correlations and correlation reproducibility, for immune metagene correlations **A.** Density plot of reproducible correlations (reproducibility *p*-value <10%) for each cancer type; **B.** Plot of densities, separated in two modes by means of the expectation–maximization algorithm; **C.** Box plot representation of the two modes in **B**; **D.** Box plot representation of correlation reproducibility *p*-values; **E.** *t*-test *p*-values of reproducible correlations across distinct cancer types, referring to the high modes shown in C; **F.** *t*-test *p*-values of reproducible correlations, comparing cancer type, referring to the low modes shown in **C**. Boxplots of reproducible correlations used the values of metagene correlations in the learning dataset (in Figures 2-4, A, excluding diagonal elements), for which correlation reproducibility had a *p*-value < 10%. Boxplots of correlation reproducibility distribution used matrices of Figures 2-5, (excluding diagonal elements).

For the purpose of the meta-analysis, we included all reproducible correlations, i.e. correlations that received a global p value of <0.1 (note that this threshold was high because there were very few correlations with reproducibility p-values <5%, as indicated in Figure 6D) and analyzed their distribution for each cancer type. The density plots shown in Figure 6A indicate clear organ-specific differences in the correlations among metagenes reflecting the cancer immune infiltrate. A finer analysis of these correlations can be obtained by separating these densities into two modes, by means of an expectation maximization algorithm (Figure 6B). In melanomas and in mammary carcinomas the higher modes of the correlation coefficient (R) were superior to those observed in colorectal cancer and in non-small cell lung cancer (Figure 6C, 6E). In mammary carcinoma, the range of correlation coefficients of the lower mode was broader than for the three other cancer types (Figure 6D, 6E). As a correlate of this finding, reproducible correlation matrices exhibited more anticorrelated subgroups in breast cancer microarrays (Figure 4G) than for all other cancer types (Figures 2G, 3G, 5G). The reproducibility of correlations among immune subtype-related metagenes also revealed organ-specific variations, as indicated by box plot analysis (Figure 6D). Thus, the reproducibility of such correlations was the highest (with hence the lowest p values), in breast cancer, followed by colorectal carcinoma, non-small cell lung cancer and melanoma. As a visual correlate of this finding, the black color (which symbolizes high reproducibility) is more preponderant in correlation reproducibility matrices describing breast cancer microarray (Figure 4F) than in other cancer types (Figures 2F, 3F, 5F).

Subsequently we extended this type of analysis beyond the immune system, by looking at metagenes that reflect endoplasmic reticulum (ER) stress, lysosomal function and autophagy, while correlating such metagenes with immune cell subtype-relevant metagents (Supplemental Figure 1). Globally, the mean R values obtained for this kind of correlation was close to zero for all cancer types (Supplemental Figure 1A). However the reproducibility of correlations was somewhat better for colorectal and mammary carcinomas than for lung cancers and melanomas (Supplemental Figure 1B). Nonetheless, there were rather few correlations between, on one hand, immune parameters and, on the other hand, cellular functions (ER stress, lysosomal function, autophagy) that reached statistical significance (*p* < 0.05), much less though than this was found within the immune cell subtype-specific metagenes (Figure 6D and Supplemental Figure 1B).

### Prognostic features of individual metagene correlations in breast cancer

Clinical information on the response of breast cancer patients to neo-adjuvant chemotherapy was available for four out of the five cohorts (Bonnefoi, Hatzis, Tabchy, Korde) analyzed in this study, allowing us to classify patients into ‘responders’ and ‘non-responders’ (Table 1). We previously reported on three of these four cohorts, showing that a high level of expression of a CXCL13-centered, highly reproducible metagene signature indicative of the intratumoral presence of interferon-γ-producing T cells had positive prognostic features, as did a number of additional immune-related metagenes [18]. We used this information to interrogate our databases with regard to the following two questions. First, are their correlations among immune cell type-related metagenes that distinguish responders and non-responders? Second, are there correlation between immune cell type-related metagenes and cell-stress related metagenes that differ among responders and non-responders?

**Figure 7:**
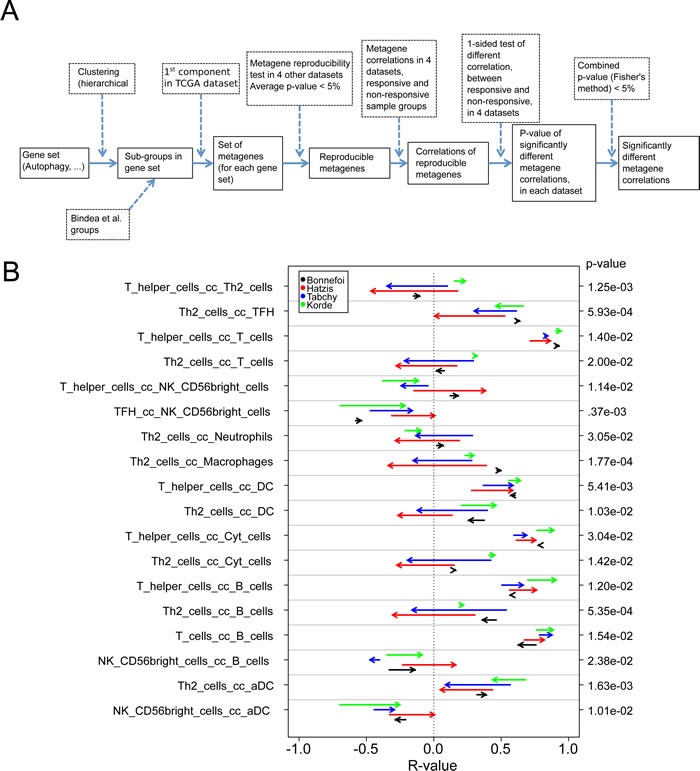

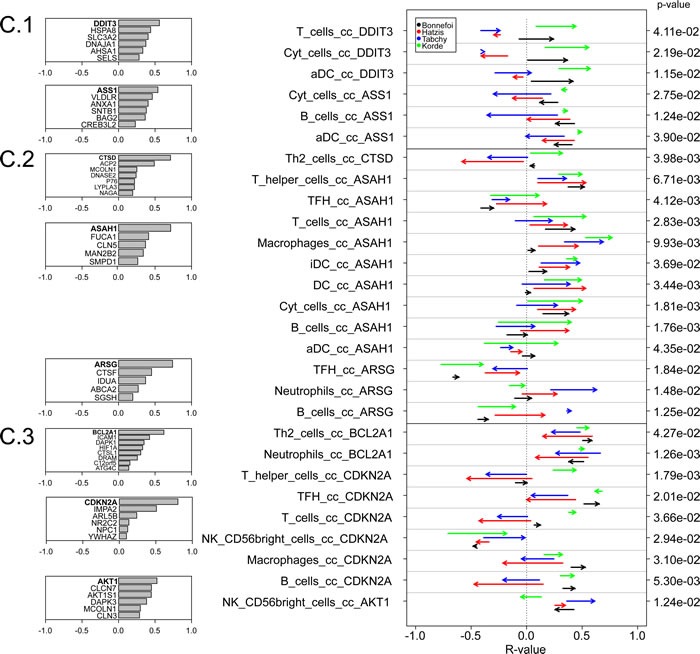
Variability of metagene correlations, upon treatment response in breast cancer **A.** Flow chart for producing B & C. (B & C) Metagene correlations, when they are significantly different upon treatment response, **B.** for immune metagenes, **C.** for correlations between immune metagenes and ER-stress (C.1), lysosome (C.2), autophagy (C.3). Heads of arrow represent correlations for responsive tumors, tails of arrow represent correlation for non-responsive tumors. *P*-values were associated to the combination of correlation difference, as delineated in A. Colors represent different datasets. On the left, details of metagene are plotted, for ER-stress, lysosomes and autophagy (the name of a metagene is defined by its most representative gene).

To respond to these questions, we followed a strategy (Figure 7A) that involved the generation of reproducible metagenes with the consequent exclusion of non-reproducible metagenes (as in Figure 1), the meta-analysis of correlations among metagenes and the subsequent exclusion of non-reproducible correlations (as in Figure 3), followed by the analysis of correlations among metagenes that differ between responders and non-responders, including only those differences that showed some degree of coherence among the four analyzable cohorts (with a combined *p*-value calculated according to Fisher's exact test of *p* < 0.05). Only very few correlations among immune cell type-related metagenes differed among responders and non-responders (Figure 7B). Thus the correlation between a metagene reflecting the intratumoral presence of Th2 cells and other leukocyte subtypes (T cells, neutrophils, macrophages, cytotoxic cells, B cells) tended to have higher R values in non-responders (start of the arrows in Figure 7B) than in responders (arrowheads in Figure 7B), although these trends were not uniform among all four cohorts.

We identified a few correlations between immune cell type-related metagenes and cell stress-related metagenes that neatly distinguished responders from non-responders (Figure 7C). Among these, one metagene (ASAH1) correlated more with a number of leukocyte subtype-related metagenes (T cells, macrophages, immature dendritic cells, neutrophils, B cells) in responders than in non-responders. This trend was uniform among all four cohorts. *ASAH1* codes for N-acylsphingosine amidohydrolase-1 (also called acid ceramidase), the enzyme that catalyzes the synthesis and degradation of ceramide into sphingosine, both of which have immunomodulatory functions [38, 39]. *ASAH1* formed a reproducible metagene with a few other lysosome-relevant genes, namely *FUCA1* (which codes for tissue alpha-L-fucosidase, which breaks down fucose), *CLN5* (coding for ceroid-lipofuscinosis neuronal protein 5), *MAN2B2* (which codes for mannosidase, alpha, class 2B, member) and *SMPD1* (coding for sphingomyelin phosphodiesterase 1, also known as acidic sphingomyelinase, ASM) (Figure 7C). This was not directly related to metagene expression levels because many leukocyte-related metagenes were overexpressed in responsive tumors, contrasting with the ASAH1 metagene that was actually underexpressed in responsive tumors (Supplemental Figure 8).

### Concluding remarks

The present study revealed that multiple metagenes initially designed for the analysis of leukocyte subtypes infiltrating colorectal cancers [11] were highly reproducible across distinct cancer cohorts, underscoring that they indeed share cell type-specific expression patterns that are stable enough to be detectable in different tissues, including the highly heterogeneous tumor microenvironment. Subsequent correlation analyses demonstrated a large number of positive correlations in the expression of distinct immune-related metagenes. However, there was a neat difference among the four cancer types included in this meta-analysis. For example, the strength of positive correlations was stronger in breast cancer and melanoma then in colon and lung carcinomas. Moreover, breast cancer represented the only type of malignancy in which multiple negative correlations were tangible and reproducible. The reproducibility of the correlations among immune-relevant metagenes was stronger in mammary carcinoma (followed by colorectal cancer, non-small cell lung cancer and melanoma, in this order), reflecting the fact that breast cancer has an intrinsically better prognosis than the three other malignancies [40]. It is tempting to translate these statistical calculations in immunological terms. Indeed, the strong and homeostatic correlations among the expression patterns of distinct immune cell subtype-specific metagenes suggest that, in breast cancer, immune infiltrates – if present – are highly organized, contrasting with other cancer types where such an organization, perhaps in tertiary lymphoid structures [5], may be less efficient, resulting in the presence of isolated (or few) lymphoid and myeloid subpopulations. Systematic histological and immunohistochemical analyses will be required to explore this speculation at the experimental level.

We were unable to detect highly consistent shifts in the correlation among immune cell subtype-relevant metagenes that would distinguish responders and non-responders among a population of breast cancer patients treated with neo-adjuvant chemotherapy. Obviously, there are large differences in the characteristics of these populations as well as in the treatment schedules (Table 1) that might render undetectable any subtle impact of such intra-immune correlations on the fate of breast cancer patients. In contrast, we found consistent shifts in the relationship between the expression level of one lysosome-relevant metagene (composed by *ASAH1*, *FUCA1*, *CLN5*, *MAN2B2* and *SMPD1*, which all code for lysosomal hydrolases with the exception of *CLN5*) and several major leukocyte populations (including T and B lymphocytes, as well as several major myeloid cell types, including immature dendritic cells, macrophages and neutrophils) from responders to non-responders [41]. In responders, these correlations tended to be more positive than in non-responders. High expression of *ASAH1* (independently of any immune parameter) is associated with positive prognosis in breast cancer [42], and the estrogen receptor antagonist tamoxifen reportedly inhibits this enzyme via an off-target effect [43]. High expression of *FUCA1* also has been attributed a positive prognostic value in breast cancer [44]. These results are based on microarray results as well, meaning that there is information available whether these genes are expressed by immune cells or by other parenchymatous and stromal elements of the tumor.

Irrespective of these uncertainties, the present study reveals novel facets of the correlations between immune- and cell stress-related metagenes. In particular, it appears that the immune system is more ‘structured’ (with a higher degree of reproducible positive correlations in the expression or leukocyte subtype-related metagenes) in the immune infiltrate of tumors with an intrinsically favorable prognosis such as breast cancer than in cancers with a poor prognosis such as non-small cell lung cancer and melanoma.

## MATERIALS AND METHODS

### Datasets

Public datasets of transcriptome microarray were considered. For each cancer type, 5 transcriptome datasets were used. Among the 5 datasets, the first one (with the largest number of samples) was used as “learning” dataset in order to construct metagenes.

For Melanoma, the following datasets were used:

“Xu” for melanomas from dataset GSE8401 (83 samples), “Harlin” for melanomas from dataset GSE12627 (44 samples), “Bogunovic” for melanomas from dataset GSE19234 (44 samples), “RikerMel” for melanomas from skin cancer dataset GSE7553 (56 samples), “Talantov” for melanomas from dataset GSE3189 (45 samples).

For Colon cancers, the following datasets were used:

“BittColon” for colon cancers from multi-cancer dataset GSE2109 (307 samples), “Smith” for colorectal cancers of dataset GSE17536 (177 samples), “Vilar1” for colon cancers of the dataset GSE26682 (first part, 155 samples), “Vilar2” for colon cancers of the dataset GSE26682 (second part, 176 samples), “TCGA” for colon cancers from TCGA consortium (174 samples).

For Breast cancers, the following datasets were used:

“TCGA” for breast cancers from TCGA consortium (522 samples), “Bonnefoi” for breast cancers from dataset GSE6861 (161 samples), “Hatzis” for breast cancers from dataset GSE25065 (198 samples), “Tabchy” for breast cancers from dataset GSE20271 (178 samples), “Korde” for breast cancers from dataset GSE18728 (61 samples). The latter four datasets were chosen because treatment response are annotated.

For Lung cancers, the following datasets were used:

“LungAC” for lung adenocarcinoma from a consortium (Director's Challenge Lung Study, National Cancer Institute (NHI)) (462 samples), “Lee” for lung adenocarcinoma and squamous cell carcinoma of the dataset GSE8894 (138 samples), “Okayama” for lung adenocarcinoma of the dataset GSE31210 (226 samples), “Raponi” for squamous cell carcinoma of the dataset GSE4573 (130 samples), “TCGA” for squamous cell carcinoma from TCGA consortium (134 samples). We joined these two lung cancer subtypes to have enough samples for statistical analysis. It should be noted that the metagene correlation matrices did not exhibit different patterns according to the distinct tumor subtypes (Figure 5).

If transcriptome were produced by Affymetrix® technology (www.affymetrix.com), the best probe sets were chosen according to “Jetset” annotation ([45]).

Datasets were downloaded and processed within the R environment [46].

For all datasets, normalized data available on the web was used. Log (base 2) was taken for those datasets that did not have Gaussian tails in their expression distribution.

### Construction of metagenes

Metagenes were constructed according to the method described in Stoll et al. [18], by identifying sets of genes and assigning coefficients inside sets:

1) Identification of sets of genes. For immune metagenes, sets were taken from Bindea et al. [11], using genes that represent immune cell types. For other metagenes, (autophagy, ER-stress and lysosome), sets of genes were extracted from genes related to autophagy, ER-stress and lysosomes [18]. Hierarchical clustering was applied to these genes in the learning dataset. Hierarchical clusters were reduced to obtain a maximum of 20 gene sets related to autophagy, ER-stress and lysosomes.

2) Assignation of gene coefficients. Inside each gene set, coefficients were obtained by performing principal component analyses on covariance of gene expression, keeping the first component, in the learning dataset. By construction, metagenes differed for each cancer type.

### Correlation of metagenes

Correlations of metagenes were obtained by calculating Pearson's correlation coefficients applied to metagene expressions. For a given sample, metagene expression was obtained as the scalar product of gene expressions and gene coefficients within the metagene. We construct a submatrix of metagene correlations for each learning dataset (Figures 2G-5G). Visual inspection of correlation reproducibility led to the identification of the subpart of the correlation matrix that was most reproducible (yellow rectangle in bottom left of Figures 2H-5H).

Pearson's correlation was chosen because each datasets have Gaussian tails in their expression distribution. Nonetheless, it has been shown [47] that low expression values in microarrays poorly correlate with expression values obtained by qPCR, which can affect the value of correlation coefficients. To test this, we considered alternative metagene correlations by removing data for genes that are poorly expressed and hence fall into the lower tail of the gene expression distribution. We used non-equality correlation tests (Steiger test in R [46], package “psych” [48]), to determine if metagene correlation coefficients depended on such outliers. We applied this procedure to the two largest Affymetrix® datasets in breast carcinoma (Bonnefoi and Hatzis), within immune metagenes. Global correlation patterns (similar to Figure 6) were stable (see Supplemental Figures 2 and 3) and almost all *p*-values associated with correlation differences were larger than 5% (see Supplemental Figures 4 and 5 for *p*-value distribution)

The cancer stage dependence was tested by means of a similar procedure. The TCGA dataset was separated into two parts encompassing either stages I-II or stages III-IV. Global correlation patterns are shown in Supplemental Figure 6, for immune metagene correlation coefficients. The difference between boxplots is smaller than those observed between different cancer types in Figure 6C. Most *p*-values associated with correlation difference are larger than 5% (see Supplemental Figure 7 for *p*-value distribution).

### Statistical tests

Metagene reproducibility was computed according to Stoll et al. [18], using a bootstrapping method that compared metagene components. By construction, a *p*-value was associated with each metagene, in each non-learning dataset.

Metagene correlation reproducibility was computed according to Stoll et al. [18], using a bootstrapping method based on correlation variance between datasets. This method is different from a more classical approach that would combine correlation *p*-values; in particular, low values of correlation that are similar within datasets produce a low *p*-value of correlation reproducibility. In addition, this bootstrapping approach is supposed to avoid bias due to the size of datasets (for instance, melanoma datasets were significantly smaller that the others).

### Selection of treatment dependent correlations

A correlation difference test was used to select correlations that vary between responsive and non-responsive tumor. It is based on the Steiger Test (r.test in R package “psych” [48]). This method is applied for a “one-sided test of different correlation, between responsive and non-responsive, in 4 datasets” in the flow chart of Figure 7A, for producing Figure 7B, 7C.

### Two modes distribution separation

The package “mixtools” [49], within R environment [46], was used to separate reproducible correlation distribution (Figure 6A) in two parts, applying an EM algorithm on a double Gaussian model. We imposed that each Gaussian mode represents at least 20% of the whole set of reproducible correlations

## SUPPLEMENTARY MATERIALS, FIGURES


