# Supplementary material for: Meta-analysis of organ-specific differences in the structure of the immune infiltrate in major malignancies
Source: Oncotarget. 2015 May 19;6(14):11894–909. doi: 10.18632/oncotarget.4180 (PMC4494912; doi:10.18632/oncotarget.4180)
Supplement: Supplementary file 1 [file oncotarget-06-11894-s001.pdf]

Meta-analysis of organ-specific differences in the structure of the immune infiltrate in major malignancies

Supplementary Material

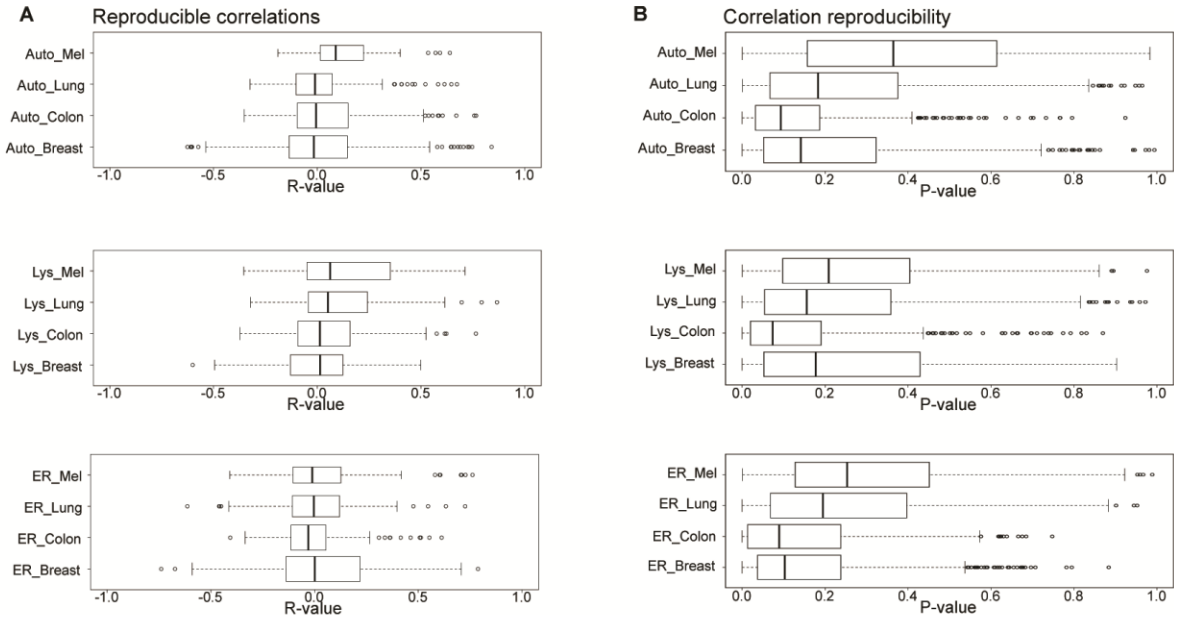

Supplemental Figure 1: Global pattern of reproducible correlations and correlation reproducibility, for correlations between immune metagenes and autophagy metagenes, correlations between immune metagenes and lysosome metagenes, correlations between immune metagenes and ER-stress metagenes.

Boxplot of reproducible correlations used the values of metagene correlations in learning dataset for which correlation reproducibility has a p-value smaller than 10%.

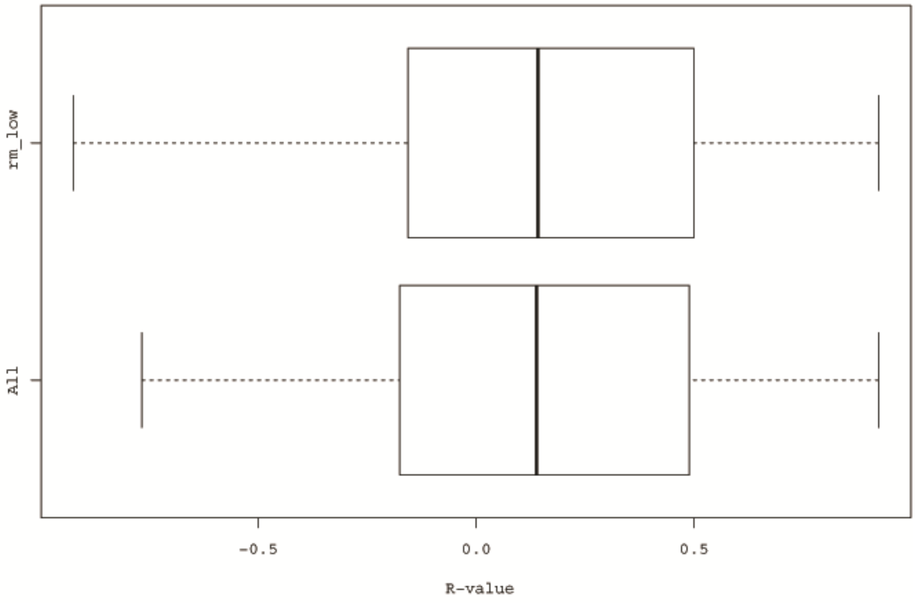

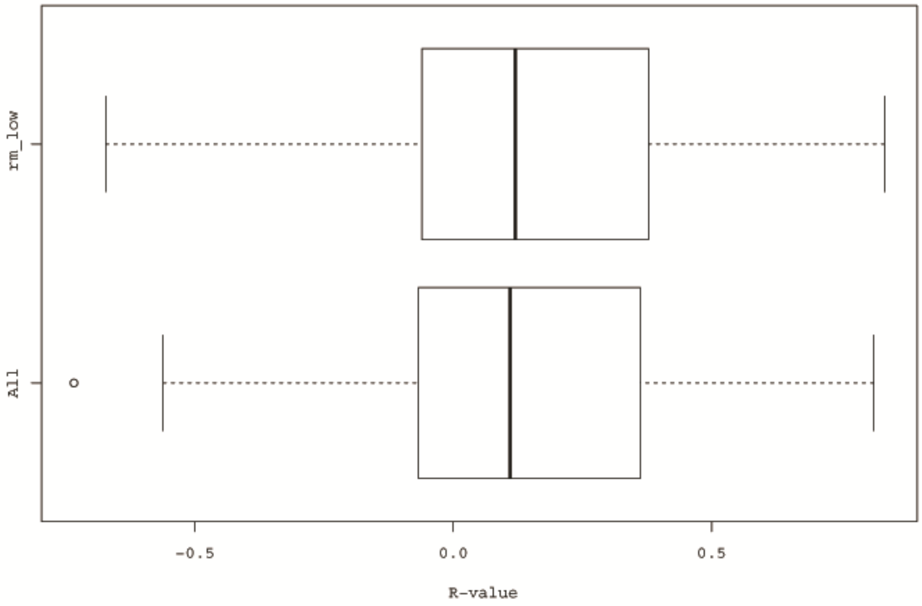

Supplemental Figures 2 and 3: (for Bonnefoi and Hatzis datasets respectively, in breast carcinoma): boxplot representation of metagene correlation coefficients, with or without low expression values.

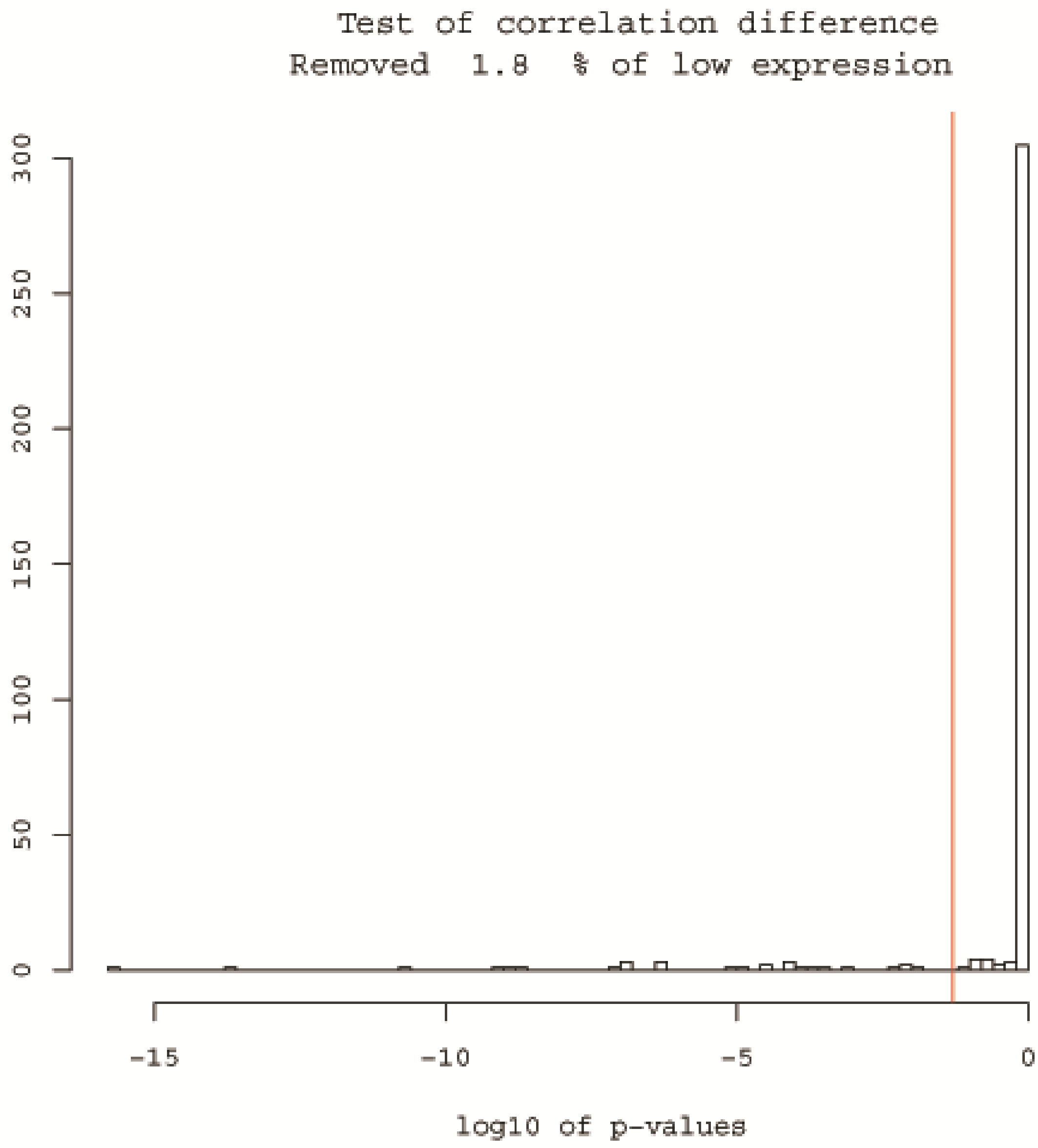

Test of correlation difference
Removed 1.8 % of low expression
300
250
200
150
100
50
0
-15
-10
-5
0
log10 of p-values

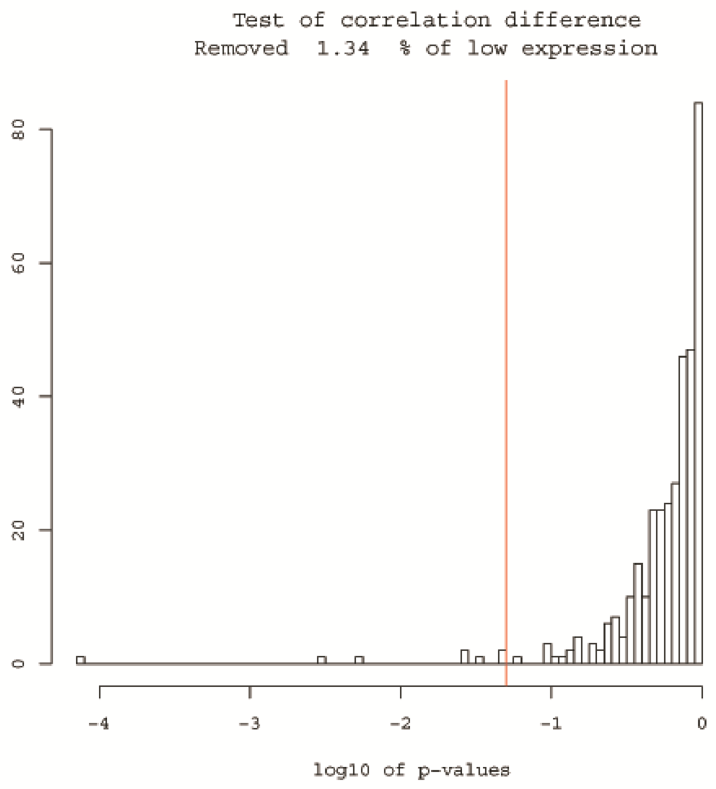

Supplemental Figures 4 and 5: (for Bonnefoi and Hatzis datasets respectively, in breast carcinoma): p-value histogram for test of correlation difference, between metagene correlation coefficients, with or without low expression values. Red line represents the value of 5%.

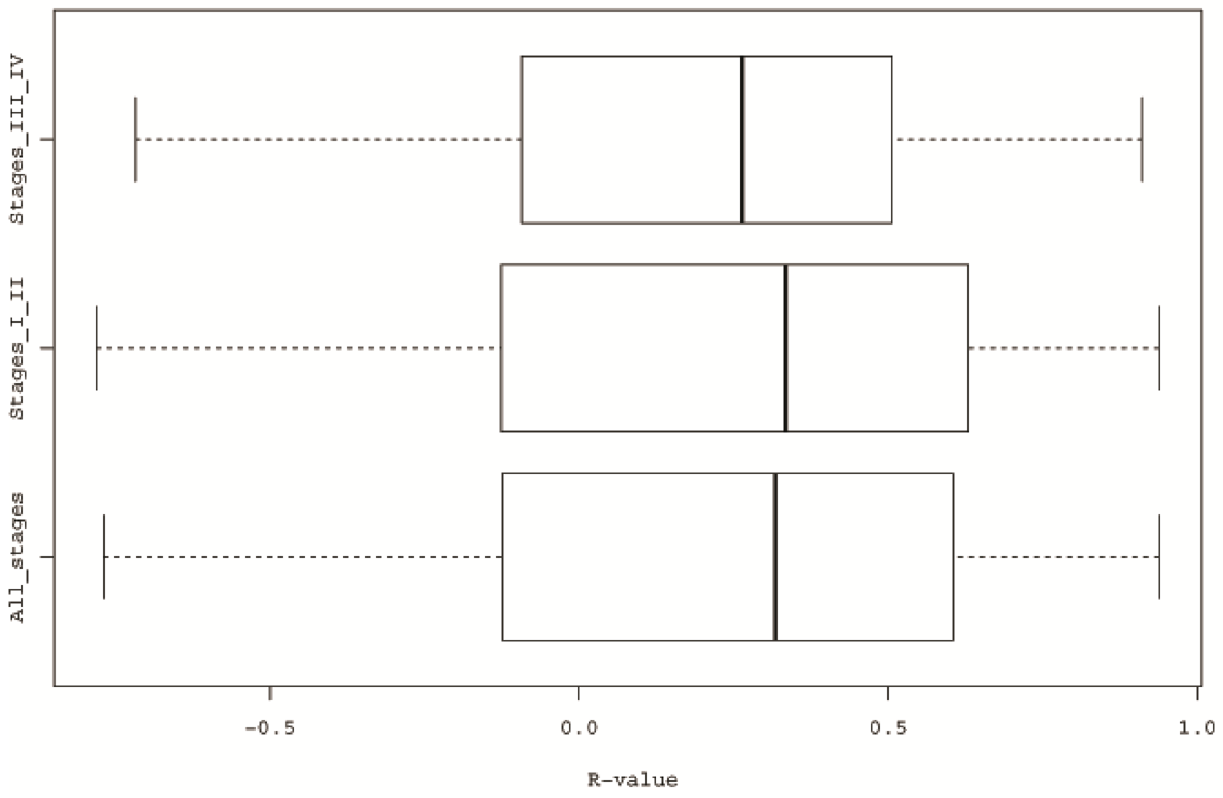

Supplemental Figure 6: (for TCGA dataset in breast carcinoma): boxplot representation of metagene correlation coefficients, for tumors of stages I-II, for tumors of stages III-IV, for all tumors.

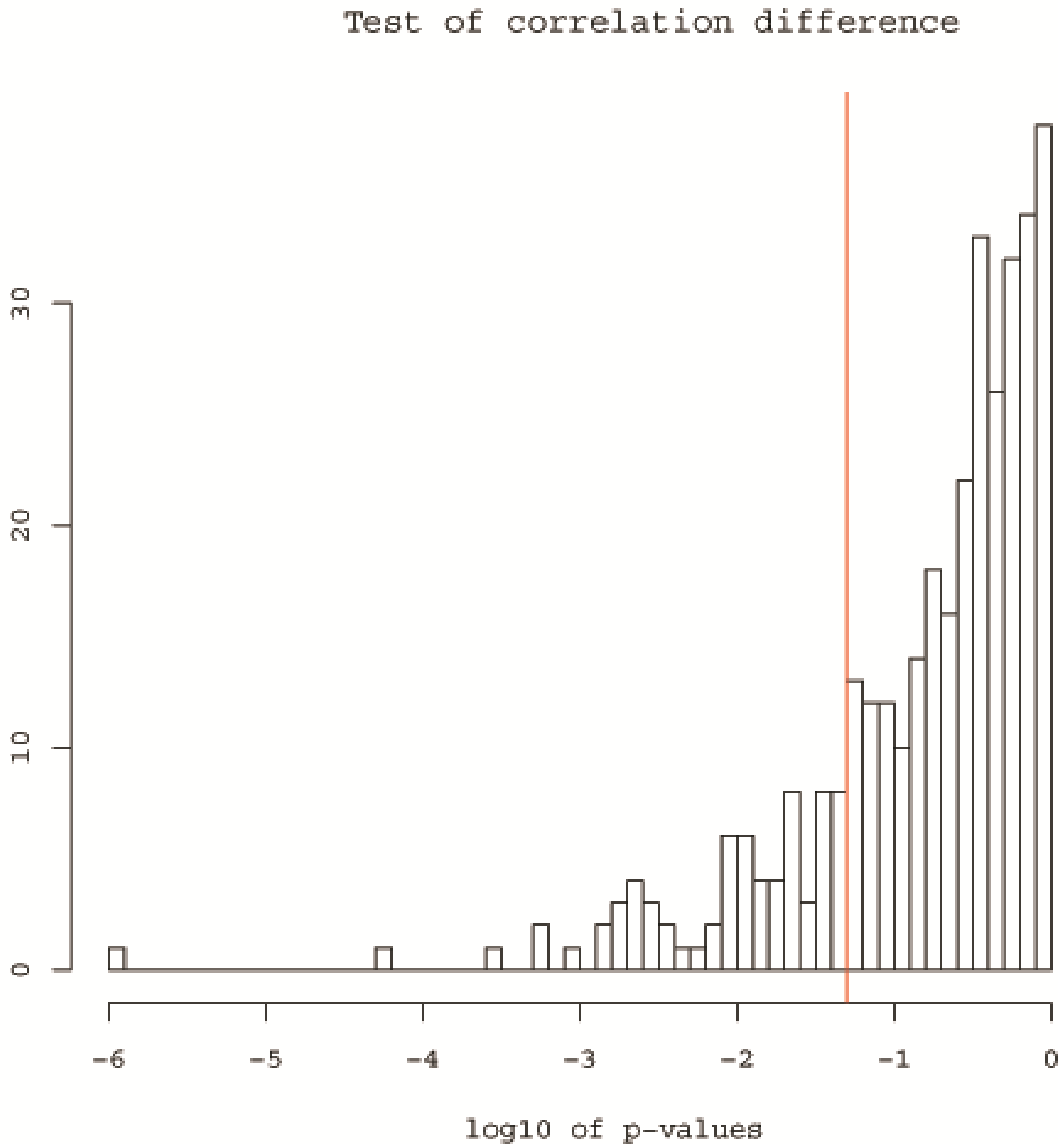

Supplemental Figure 7: (for TCGA dataset in breast carcinoma): p-value histogram for test of correlation difference, between metagene correlation coefficients, for tumors of stages I-II compared to tumors of stages III-IV. The red line represents the value of 5%.

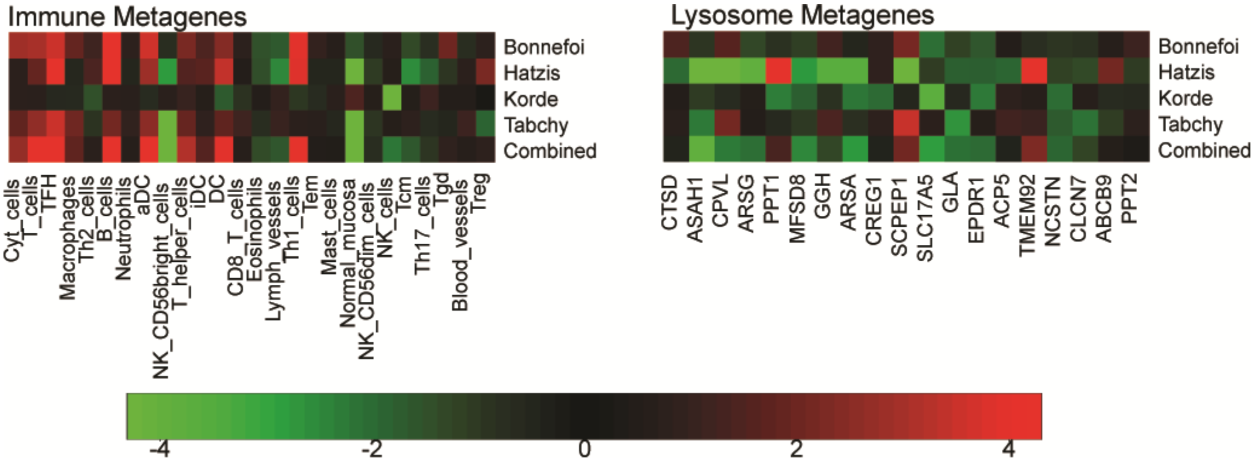

Supplemental Figure 8: Signed log of t-test p-values, comparing metagenes expression in responsive breast tumors vs non-responsive breast tumors. Immune and lysosome-related metagenes were considered. Positive signs (red) indicate overexpression in responsive tumors, while negative signs (green) indicate overexpression in non-responsive tumors. The four breast cancer datasets (excluding the learning one, TCGA) were considered. The combined p-value was computed by Fisher's method applied on one-sided t-tests.
